# Comparisons of Functional Properties of Polysaccharides from *Nostoc flagelliforme* under Three Culture Conditions

**DOI:** 10.3390/polym11020263

**Published:** 2019-02-04

**Authors:** Shi-Gang Shen, Ya-Hui Lin, Dong-Xue Zhao, Yi-Kai Wu, Rong-Rong Yan, Hua-Bing Zhao, Zhi-Lei Tan, Shi-Ru Jia, Pei-Pei Han

**Affiliations:** 1 State Key Laboratory of Food Nutrition and Safety, Key Laboratory of Industrial Fermentation Microbiology, Ministry of Education, College of Biotechnology, Tianjin University of Science and Technology, Tianjin 300457, China; ssg540@163.com (S.-G.S.); linyahui@mail.tust.edu.cn (Y.-H.L.); zhaodongxue@mail.tust.edu.cn (D.-X.Z.); wuyikai@mail.tust.edu.cn (Y.-K.W.); yanrongrong@mail.tust.edu.cn (R.-R.Y.); zhaohuabing@tust.edu.cn (H.-B.Z.); 2 Tianjin Key Laboratory for Prevention and Control of Occupational and Environmental Hazards, Logistics University of Chinese People’s Armed Police Forces, Tianjin 300309, China

**Keywords:** functional properties, *nostoc flagelliforme*, culture condition, polysaccharides

## Abstract

*Nostoc flagelliforme* is an edible cyanobacterium with excellent food and herbal values. It has been used as food in China for more than 2000 years. Many studies have been focused on improving the yield and bioactivity of *Nostoc flagelliforme* polysaccharides although these have ignored the functional properties. In this study, we extracted and purified three polysaccharides (WL-CPS, NaCl-CPS and Glu-CPS) from *Nostoc flagelliforme* under normal, salt stress and mixotrophic culture conditions, respectively, in order to change the physicochemical properties of polysaccharides with the aim of obtaining better functional properties. Both salt stress and mixotrophic culture conditions increased the specific yield of polysaccharides. Their functional properties were comparatively investigated and the results showed that NaCl-CPS exhibited the highest emulsification activity and flocculation capability, which was also higher than that of some commercial products. In contrast, Glu-CPS exhibited the highest water and oil holding capacities, foaming property, intrinsic viscosity and bile acids binding capacity. Our results indicated that both NaCl-CPS and Glu-CPS could be considered to be functional polysaccharides according to their respective characteristics, which have great potential in numerous applications, such as food, pharmaceutical, cosmetic, chemical and mineral industries. These findings also demonstrated the potential application of the proper regulation of culture conditions in the development of polysaccharides with desired functional properties.

## 1. Introduction

In the last few decades, polysaccharides have emerged as an important class of biopolymers due to their potential applications in different fields of applied and industrial biotechnology, such as in the removal of heavy metals from wastewater or being used as thickening or gelling agents, food additives and drug candidates for many therapies [1,2,3,4]. Moreover, polysaccharides derived from natural sources are generally considered to have low toxicity and have long been used, particularly in food products because consumers prefer natural additives [5]. They are increasingly incorporated in cooked products in order to enhance cooking yield, improve water holding capacity, modify texture, reduce formulation costs and improve the stability of emulsions. In addition, many of these polysaccharides are able to form gels or viscous solutions that are of great industrial value. These applications of polysaccharides are attributed to their physicochemical properties [6,7], which depend on their structural features, such as molecular weight, monosaccharide composition and sequence of linkages [8,9,10]. Due to their potential applications in functional foods, a growing number of studies have focused on the extraction of polysaccharides from a variety of microorganisms and plants [11,12,13].

*Nostoc flagelliforme* is an edible cyanobacterium with excellent food and herbal values, which has been used as food in China for more than 2000 years. The extracellular polysaccharides (EPS) of *N. flagelliforme*, which are either loosely attached to the cell surface or released into the surrounding environment, have been proved to possess antiviral, antioxidant and anti-tumor activities [14,15]. *N. flagelliforme* also produces capsular polysaccharides (CPS) that are strongly bound to the cell surface, which have been found with similar biological activities to the EPS [15,16], which makes *N. flagelliforme* a promising resource for the development of functional foods. However, many studies have focused on increasing the yield of polysaccharides and improving their bioactivities [17,18] while ignoring the functional properties of polysaccharides.

It is reported that *Nostoc flagelliforme* polysaccharides have high intrinsic viscosity, good emulsification activity and excellent flocculation capability [19]. However, there is no systematic study on the functional properties of *Nostoc flagelliforme* polysaccharides. To fully utilize the polysaccharides, it is necessary to develop a simple and reliable method to control the quality of polysaccharides. To investigate the effect of culture conditions on the physicochemical properties and antioxidant activities of polysaccharides from *Nostoc flagelliforme*, three polysaccharides were extracted and purified [17]. It was found that these polysaccharides all had different sugar and uronic acid contents and monosaccharide composition as well as average molecular weights. Thus, they showed significant differences in their antioxidant activities [17]. Moreover, it was also observed that the antioxidant activity of polysaccharides could be greatly enhanced by proper regulation of culture conditions [17]. Based on these findings, a strategy for regulating the culture conditions is proposed to change the physicochemical properties of polysaccharides with the aim of obtaining better functional properties. Therefore, in this study, the effects of different culture conditions, including salt stress and mixotrophic culture, on the functional properties of polysaccharides were comprehensively investigated.

## 2. Materials and Methods

### 2.1. Strains and Culture Conditions

Tianjin Key Lab of Industrial Microbiology (Tianjin, China) provided the *Nostoc flagelliforme* cells (TCCC11757), which were cultured in the BG-11 medium [20]. Inocula were prepared regularly (10 days) in 500-mL Erlenmeyer flasks at 25 °C under continuous cool-white fluorescent light illumination of 60 μmol photons m^−2^ s^−1^. After this, it was inoculated (10%, *v*/*v*) to an 80-L airlift photo-bioreactor with 60 L of BG-11 medium at 25 °C. The aeration rate was set to 0.8 vvm and the light intensity was 60 μmol photons m^−2^ s^−1^, which was provided by 8 fluorescent lamps set into the bioreactor. The experiment was divided into three groups: control group (cells were cultured in BG-11 medium for 24 days), salt stress group (cells were first cultured in BG-11 medium for 15 days, before NaCl was added to a final concentration of 0.5 mol/L and continued for culturing 9 days) and mixotrophic culture group (cells were first cultured in BG-11 medium for 15 days, before glucose was added to a final concentration of 4 g/L and continued for culturing 9 days).

### 2.2. The Measurement of Cell Growth and CPS Production

The growth increment of the *Nostoc flagelliforme* cells was measured with the dry mass (DW) method. A total of 40 mL of the culture medium was centrifuged in a 50-mL centrifuge tube at 4000× *g* for 10 min. The supernatant was removed and the tube (preweighed) with the pellet was washed twice with distilled water before being dried at 80 °C until the weight was constant. The measurement of CPS production was obtained as previously reported [13].

### 2.3. Isolation and Purification of Polysaccharides

The *N. flagelliforme* culture was centrifuged at 10,000× *g* for 10 min at 4 °C to harvest cells, which were further processed for the extraction of CPS. The crude CPS was obtained by extracting lyophilized cells in hot water at 80 °C for 6 h, before this was concentrated by ultra-filtration. The concentrate was added to 80% (*v*/*v*) alcohol and kept for 12 h at 4 °C. Crude CPS was collected by centrifuging and subsequent freeze-drying. The crude CPS was then redissolved in deionized water and further purified according to the following steps. The solution was added to a DEAE-650M cellulose anion exchange column (Φ 2.6 cm × 60 cm) (TOSOH Corporation, Tokyo, Japan) before being processed with a Sephadex G100 gel chromatographic column (Φ 1.6 cm × 80 cm) (Pharmacia Corporation, Stockholm, Sweden). The first column was eluted with distilled water, which was followed by a linear gradient of 0–1.0 mol/L NaCl solution. The second column was eluted with distilled water. Their flow rate was 1.0 and 0.5 mL/min, respectively. The main fractions were collected and the carbohydrates were monitored via the phenol-sulfuric acid method [21]. The purified CPS was obtained by merging the carbohydrate-positive fractions, dialysis and freeze-drying. Finally, three polysaccharides named WL-CPS, NaCl-CPS and Glu-CPS were obtained.

### 2.4. Determination of Functional Properties

The functional properties of WL-CPS, NaCl-CPS and Glu-CPS were obtained by determining the intrinsic viscosity, thermal stability, water holding capacity, oil holding capacity, foaming properties, emulsifying and flocculating properties and the bile acids binding capacity.

#### 2.4.1. Intrinsic Viscosity Analysis

The intrinsic viscosity of the polysaccharide samples was measured according to the previously reported method [22]. Briefly, 15 mL of a polysaccharide solution (0.1 g/L) was placed in an ubbelohde capillary tube (typeΦ 0.5 mm, Equity Instrument Factory, Shanghai, China) and immersed in a thermostat bath at 25 °C. This method was determined by the measurement of descent time (t) of polysaccharides solution flowing through the capillary tube. Distilled water was used to determine t_0_. The relative viscosity (η_r_) was determined from η_r_ = t/t_0_ and the specific viscosity (η_sp_) was obtained from η_sp_ = (t − t_0_)/t_0_. The intrinsic viscosity (η) was determined using the following equation:

[η] = (η_sp_ + 5ln η_r_)/6c (1)

where t is the polysaccharide solution flow time; t_0_ is the distilled water flow time; and c is the concentration of the polysaccharide solutions.

#### 2.4.2. Thermogravimetric Analysis (TGA)

Each polysaccharide sample was analyzed by TGA using a Q5000IR thermogravimetric analyzer (TA Instruments, Newcastle, DE, USA). Approximately 10 mg of each sample was placed in a sample pan and heated from 25 to 800 °C at 10 °C /min under nitrogen atmosphere.

#### 2.4.3. Water-holding Capacity (WHC) and Oil-holding Capacity (OHC)

The WHC and OHC of polysaccharides samples were measured according to the previous method with slight modifications [23]. The samples (50 mg) were dispersed in 5 mL of distilled water or 5 mL of peanut oil to determine WHC and OHC, respectively. The mixture was stirred and left at room temperature for 1 h before being centrifuged at 4800× *g* for 10 min. The upper phase was removed carefully and the centrifuge tube was drained for 30 min on a filter paper. The ratio between the weight of the tube content after draining and the weight of the polysaccharides samples was determined and the capacity (%) was reported as grams of water or oil bound per gram of the polysaccharides on a dry basis.

#### 2.4.4. Foaming Properties

Foam capacity (FC) and foam stability (FS) were determined according to the reported protocol [24]. Briefly, 5 mL of polysaccharide solution at a concentration of 0.5% (*w*/*v*) was homogenized in a centrifuge tube for 5 min using vortex at room temperature. FC and FS were calculated as follows:

FC (%) = (*V*_T_ − *V*_0_)/*V*_0_ × 100 (2)

FS (%) = (*V*_t_ − *V*_0_)/*V*_0_ × 100 (3)

where *V*_T_ is the total volume after whipping; *V*_0_ is the volume before whipping and *V*_t_ is the total volume after the solution is left at room temperature for 30 min.

#### 2.4.5. Emulsifying and Flocculating Properties

The emulsifying and flocculating properties of polysaccharides were assayed according to the previously reported method [19].

#### 2.4.6. Determination of Bile Acids Binding Capacity

The in vitro ability of polysaccharides to bind bile acids in a simulated intestinal environment was determined according to the previously reported method with some modifications [25]. Briefly, 1 mL of the polysaccharide solution (0.2, 0.5 and 1.0 mg/mL) and 1.25 mL of 0.01 mol/L HCl were mixed and incubated at 37 °C for 1 h. After this acidic incubation, which simulated gastric digestion, the pH of sample was adjusted to 6.3. During the next step, 2 mL of the bile acids solution (0.72 μmol/mL) was added and mixed, followed by 2.5 mL of porcine pancreatin (10 mg/mL) that provided amylase, protease and lipase for digestion. This was subsequently incubated for 1 h at 37 °C in shaker bath. The mixtures were transferred to 10-mL centrifuge tubes and centrifuged at 4800× *g* for 10 min at 25 °C. To determine the total amount of bile acids bound by the polysaccharides, the final bile acids content in the supernatant was measured. The bile acids were measured by an Automatic Biochemical Analyzer (AU2700, Olympus, Hamburg, Germany). Values were determined from a standard curve obtained using the tested bile acids solution. Cellulose, a non bile acid binding fiber, was the negative control and cholestyramine, a bile acid binding anionic resin, was the positive control.

### 2.5. Statistical Analysis

All the experiments were performed in triplicate and all experimental data were presented as the mean ± standard deviations (SD). The data were analyzed by independent-samples t-test using the SPSS statistical software (version 20.0, IBM company, Chicago, IL, USA). Values of *P* < 0.05 were considered to be significant.

## 3. Results and Discussion

It is reported that microalgal culture conditions are the main factors affecting the biomass accumulation, polysaccharide production and structural features [12,13]. Our previous work investigated the effect of culture conditions on the physicochemical properties and antioxidant activities of polysaccharides from *Nostoc flagelliforme*. Three polysaccharides (WL-CPS, NaCl-CPS and Glu-CPS) were extracted and purified from *Nostoc flagelliforme* under normal, salt stress and mixotrophic culture conditions, respectively. When investigating their physicochemical properties, it was found that these polysaccharides had different sugar and uronic acid contents and they were mainly composed of mannose (19.80%, 20.24% and 20.81%), galactose (23.71%, 22.62% and 24.34%), glucose (46.57%, 41.07% and 39.03%) and glucuronic acid (4.00%, 4.97% and 4.04%). Furthermore, they contained trace amounts of xylose, arabinose, ribose, rhamnose, fucose and fructose. Their average molecular weights were 1.02 × 10^3^, 1.12 × 10^3^ and 1.33 × 10^3^ kDa, respectively. Moreover, the closely related results were presented in supplementary materials (Table S1).

### 3.1. Biomass and CPS Production

**Figure 1:**
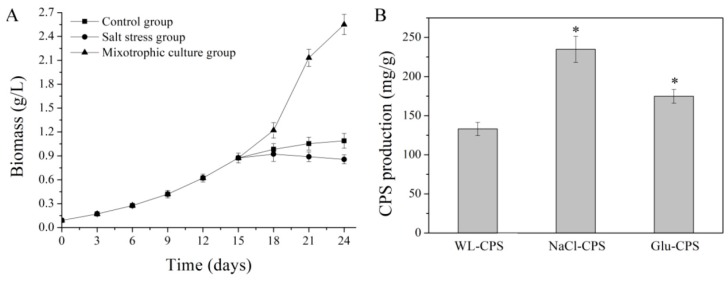
Effects of culture conditions on the biomass (**A**) and CPS (**B**) production. * Statistically different from the WL-CPS (*P* < 0.05).

In this study, the effects of culture conditions on *N. flagelliforme* culture were evaluated by comparing the biomass accumulation and CPS production. As presented in Figure 1A, after 24 days of culture, compared with the control group, the biomass of salt stress group decreased by 21.42%, while the biomass of mixotrophic culture group increased by 134.23%. In addition, both CPS productivities were significantly enhanced in the salt stress group and mixotrophic culture group (*P* < 0.05). The highest CPS production of 234.82 mg/g was achieved in the salt stress group, which was higher by 76.21% compared with control group (Figure 1B).

### 3.2. Intrinsic Viscosity

**Figure 2:**
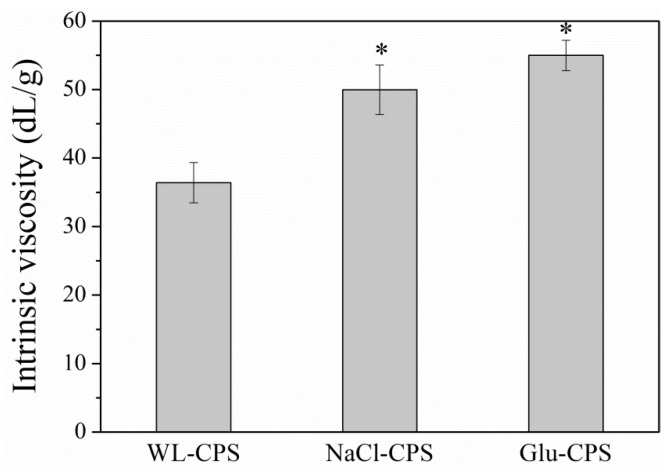
Intrinsic viscosity of *N. flagelliforme* polysaccharides. * Statistically different from the WL-CPS (*P* < 0.05).

The intrinsic viscosity is a characteristic property of a single macromolecule in a given solvent and is a measure of the hydrodynamic volume occupied by the polymer itself, which depends on the polymer’s molecular weight, chain rigidity and type of solvent. The intrinsic viscosities (η) of WL-CPS, NaCl-CPS and Glu-CPS in deionized water at 25 °C were 36.41, 49.96 and 54.97 dL/g, respectively (Figure 2). The intrinsic viscosity of Glu-CPS was the highest and the WL-CPS was the lowest, but it still was much higher than that of the polysaccharides of *N. carneum* (6.9 dL/g), *Oscillatoria* sp. (12.1 dL/g) and *Nostoc* sp. (18.4 dL/g) [26]. These results showed that different culture conditions affected the intrinsic viscosity of polysaccharides, which might be caused by the structural changes in the polysaccharides. The high intrinsic viscosity of Glu-CPS suggests that it has a strong thickening ability and a lower concentration can be used to achieve the required viscosity. On the other hand, it also suggests that the ability of polysaccharides to increase the viscosity of intestinal contents may contribute to their cholesterol-lowering effects [27].

### 3.3. Thermal Characteristic Analysis

**Figure 3:**
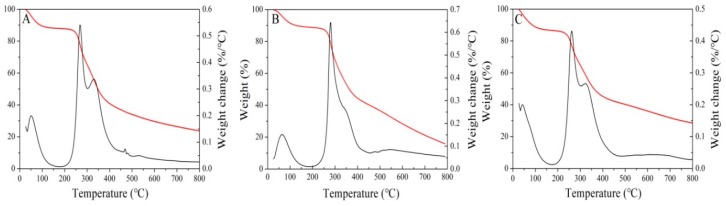
The thermogravimetric analysis of *N. flagelliforme* polysaccharides. (**A**–**C**) represent the WL-CPS, NaCl-CPS and Glu-CPS, respectively.

The thermal potential of polysaccharides, such as heat flow rate, is an important criteria for their selection and application in food industry. As shown in Figure 3, it could be clearly seen that the mass changes in polysaccharides during the heating process were mainly divided into three stages. The first stage was related to the loss of absorbed and structural water in the polysaccharides. The second stage involved the degradation of polymers, which corresponded to the depolymerization of the polysaccharide structures [28]. Finally, the third mass loss was related to the oxidation of organic matter [29]. The weight losses were 76.28%, 71.48% and 84.19% for WL-CPS, NaCl-CPS and Glu-CPS at 800 °C, respectively. The weight loss of NaCl-CPS was the lowest and it showed the strongest stability. The above results demonstrated that NaCl-CPS was more suitable to be used as a supplement in some hot foods than WL-CPS and Glu-CPS.

### 3.4. WHC and OHC of Polysaccharides

WHC and OHC are among the most popular functional properties in food processing, which are closely related to texture through the interactions between components, including water and oil.

**Figure 4:**
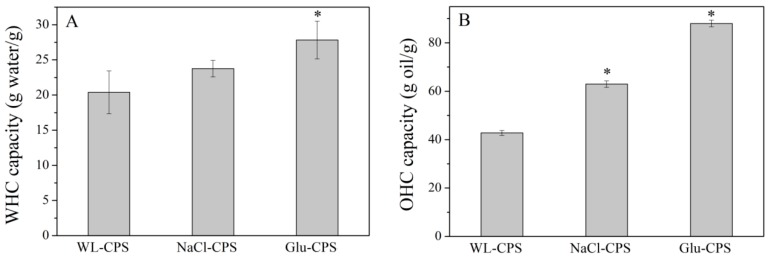
The water-holding capacity (**A**) and oil-holding capacity (**B**) of polysaccharides. * Statistically different from the WL-CPS (*P* < 0.05).

WHC is defined as the amount of water that is retained by 1 g of dry polysaccharides. It is the sum of bound water, hydrodynamic water and physically trapped water [30]. As shown in Figure 4A, Glu-CPS showed the highest WHC with a value of 27.82 *g*/*g*, which increased by 36.44% compared with the control. This value was significantly higher than the results obtained for the sulfated polysaccharides from red algae *Gigartina pistillata* (10.22 *g*/*g*) [31], seagrass *Cymodocea nodosa* (10.73 *g*/*g*) [32] and brown algae *Laminaria digitata* (17.4 *g*/*g*) [33]. Polysaccharides with high WHC could help to avoid syneresis and modify the viscosity and texture of some formulated foods [34].

OHC is defined as the amount of oil retained by the polysaccharides. From Figure 4B, Glu-CPS also showed the highest OHC with the value of 87.97 *g*/*g*, which was 2.06-fold higher than the control. Furthermore, this value was much higher than those polysaccharides from red algae *Gigartina pistillata* (1.32 *g*/*g*) [31], brown algae *Laminaria digitata* (13.8 *g*/*g*) [33] and *Ulva prolifera* (15.09 *g*/*g*) [35]. Polysaccharides with high OHC allow the stabilization of high fat food products and emulsions [34]. Furthermore, it is believed that the bound fat has less absorption in vivo and is excreted in feces [36]. The ability of polysaccharides to directly bind fat may contribute to its overall hypolipidemic activity.

It has been reported that WHC is related to pore size and the capillarity of the molecule conformational structure and polysaccharide source [37]. As for OHC, it is partially related to the chemical composition, but it is more closely linked to the porosity of the fiber structure than to the affinity of the fiber molecule to oil [38]. From Table S1, we found that both the WHC and OHC of polysaccharides increased with molecular weight, indicating that molecular weight was also an important factor related to WHC and OHC.

### 3.5. Foaming Properties of Polysaccharides

The foaming property is a surface property defined by its size and stability. Food macromolecules, such as protein and polysaccharides, play an important role in the stabilization of foams [39]. There is large demand for these macromolecules in food and cosmetic industries in order to obtain products with better consistence and sensorial quality [23]. Polysaccharide is generally used as a thickener and stabilizer in food foams due to its predominantly hydrophilic characteristics [30].

**Figure 5:**
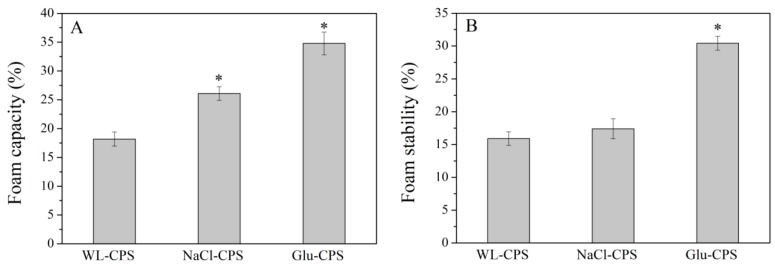
The foam capacity (**A**) and foam stability (**B**) of polysaccharides. * Statistically different from the WL-CPS (*P* < 0.05).

The foam capacity (FC) and foam stability (FS) of WL-CPS, NaCl-CPS and Glu-CPS are presented in Figure 5. The foaming properties of polysaccharides were highly dependent on culture conditions. It was likely that the foaming properties were strongly related to the molecular weight of polysaccharides. A higher molecular weight showed better foaming properties. As presented in Table S1, Glu-CPS had the highest molecular weight and therefore had best FC (34.78%) and FS (30.43%). The FC and FS of Glu-CPS was similar to that of the pectin extracted from *Opuntia ficus indica cladodes* (FC 30% and FS 25%) at the same concentration [23]. The high FC and FS of Glu-CPS can be attributed to its ability to increase the viscosity of the aqueous phase and to create a network that stabilizes the interfacial film (air-water). This result indicates a promising application of polysaccharides from different culture conditions for the improvement of foaming properties in different food formulations.

### 3.6. Emulsifying Properties of Polysaccharides

**Figure 6:**
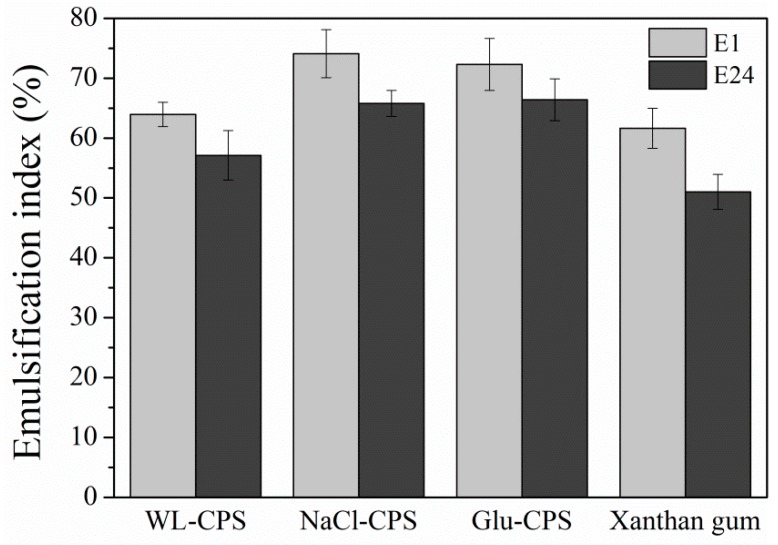
Emulsion properties of *N. flagelliforme* polysaccharides. E1 and E24 represent emulsification index after 1 h and 24 h, respectively. * Statistically different from the Xanthan gum (*P* < 0.05).

Emulsion is defined as a mixture of two normally immiscible liquids. Figure 6 shows the emulsion properties that include the emulsion capacity (EC) and emulsion stability (ES) of polysaccharides at a concentration of 0.5%. It was shown that there was no significant difference in the EC of WL-CPS compared with xanthan gum (*P* > 0.05), but the EC of NaCl-CPS and Glu-CPS were significantly higher than xanthan gum (*P* < 0.05). NaCl-CPS exhibited the highest EC, followed by Glu-CPS and WL-CPS after 1 h. The emulsifying ability of polysaccharides depends on the amount of protein linked to their structure [40]. Moreover, the presence of hydrophobic groups in the composition of deoxysugars (i.e., fucose and rhamnose) positively affected the emulsion properties [19]. From Table S1, we found that among WL-CPS, NaCl-CPS and Glu-CPS, NaCl-CPS had the highest protein content and fucose and rhamnose, which could explain its higher EC.

The ES of WL-CPS, NaCl-CPS and Glu-CPS decreased after 24 h and reached about 57.12%, 65.78% and 66.39%, respectively. This property was related to hydrophobic compounds and the size of the droplets in an emulsion based on food products [5]. In addition, the emulsion stability was proportional to the molecular weight and the uronic acid concentration [41]. ES had a positive effect on its physical stability, such as flocculation, gravitational separation and coalescence [5]. The emulsifying ability of NaCl-CPS in stabilizing emulsions might promote its applications as a bioemulsifier in numerous industrial areas.

### 3.7. Flocculating Activity of Polysaccharides

**Figure 7:**
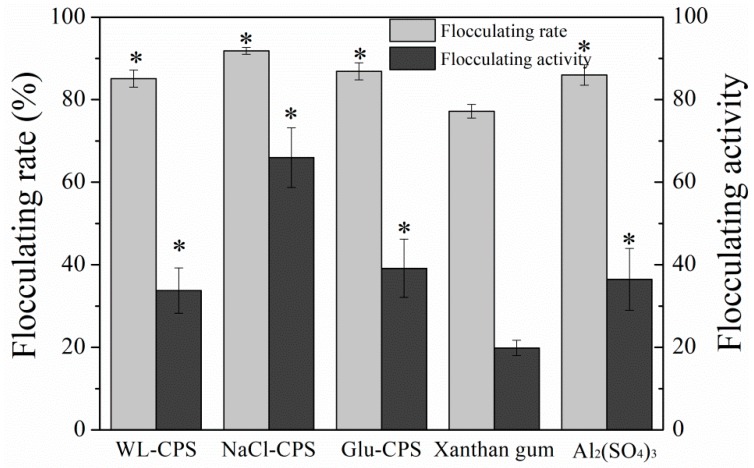
The effects of culture conditions on flocculation rate of polysaccharides using kaolin suspension at pH of 7.0. * Statistically different from the Xanthan gum (*P* < 0.05).

Flocculants are classified as inorganic flocculants, synthetic organic flocculants and bioflocculants from biological resources, which are widely used in the chemical and mineral industries, wastewater treatment and food and fermentation processes. The flocculating activity of polysaccharides from different culture conditions in the kaolin suspension was compared with other commercial flocculants (Al_2_(SO_4_)_3_ and xanthan gum). These preliminary tests were performed at room temperature and neutral pH with the flocculant concentration of 0.5 mg/L in kaolin suspension. As shown in Figure 7, NaCl-CPS had the highest flocculating activity (65.95) and flocculating rate (91.81%), followed by Glu-CPS (flocculating activity of 39.15 and flocculating rate of 86.84%) and Al_2_(SO_4_)_3_ (flocculating activity of 3.3 and flocculating rate of 79.8%). Xanthan gum had the lowest flocculating activity (19.86) and flocculating rate (77.19%). These results indicate that the polysaccharides have the high flocculating activity, especially NaCl-CPS, which envisages its potential use as a bioflocculant for colloid and cell aggregation in several applications, such as water treatment, food and mining industries and industrial downstream processing.

Our previous study found that bridging might be the flocculation mechanism of acid polysaccharides [19]. That is because the presence of uronic acid confers an overall negative charge to the polymer surface, which makes the polymers adsorb to the particles’ surface and thus promotes the formation of flocs. Among the three polysaccharides, NaCl-CPS had the maximum uronic acid (Table S1) and therefore, it showed the highest flocculating activity and flocculating rate, which corresponded to the flocculation mechanism of acid polysaccharides.

### 3.8. Bile Acids Binding Capacity

It has been reported that the solution viscosity correlates to the bile acids capacity of polysaccharides. Thus, the bile acids binding capacities of WL-CPS, NaCl-CPS and Glu-CPS in vitro were measured to evaluate the potential lipid-lowering ability [27,42]. Cholestyramine was used as a positive control in this study, which was a useful hypocholesterolemic agent for increasing bile acid excretion and decreasing cholesterol absorption [43]. Because the bile acids binding abilities of cholestyramine at a concentration of less than 1 mg/mL were particularly low and the solubility of WL-CPS, NaCl-CPS and Glu-CPS was less than 5 mg/mL, cholestyramine and cellulose at a concentration of 10 mg/mL were selected as the positive control and negative control, respectively. The results pertaining to bile acids binding capacity of polysaccharides are presented in Table 1. As expected, the cholestyramine were able to bind bile acids significantly more and cellulose significantly less than the various polysaccharides tested (*P* < 0.05). It was observed that the bile acids binding abilities of the polysaccharides at different concentrations were not significantly different (*P* > 0.05). Although all these values were significantly (*P* < 0.05) lower than that of the cholestyramine (0.367 μmol), the results showed that these polysaccharides still possessed 68.66–79.29% of the bile acids binding ability of the cholestyramine. Consistent with the previous reported observations [44], the ability of polysaccharides to bind bile acids was not proportional to their concentrations, whereas it might be related to their anionic, cationic, physical and chemical structure. Moreover, the high viscosity of the polysaccharides might also have some effects on hydrodynamic restrictions resulted in the bile acid binding ability [45]. From the above results, the polysaccharides of *N. flagelliforme* possessed high intrinsic viscosity and therefore had good bile acid binding ability.

In conclusion, both salt stress and mixotrophic culture conditions increased the specific yield of polysaccharides. Moreover, the polysaccharides from *Nostoc flagelliforme* have excellent functional properties while NaCl-CPS exhibited the highest emulsification activity and flocculation capability, which was also higher than that of some commercial products. In contrast, Glu-CPS exhibited the highest water and oil holding capacities, foaming property, intrinsic viscosity and bile acids binding capacity. The results indicated that both NaCl-CPS and Glu-CPS could be considered to be functional polysaccharides according to their respective characteristics. Furthermore, these findings demonstrated that the proper regulation of culture conditions could improve the functional properties of polysaccharides, which could also be easily applied to the culture of other microalgae species.

## Figures and Tables

**Table 1:** Bile acids binding abilities of the polysaccharides prepared from *N. flagelliforme.*

Treatment		Bile Acid Bound (μmol)	Relative Bile Acids Binding ability (%) ^a^
WL-CPS (mg/mL)	0.2	0.253 ± 0.02 *	68.94 ± 4.72 *
	0.5	0.267 ± 0.01 *	72.75 ± 1.78 *
	1.0	0.271 ± 0.01 *	73.84 ± 3.24 *
NaCl-CPS (mg/mL)	0.2	0.252 ± 0.01 *	68.66 ± 3.83 *
	0.5	0.262 ± 0.01 *	71.39 ± 1.77 *
	1.0	0.274 ± 0.01 *	74.66 ± 2.06 *
Glu-CPS (mg/mL)	0.2	0.267 ± 0.01 *	72.75 ± 3.54 *
	0.5	0.271 ± 0.01 *	73.84 ± 2.65 *
	1.0	0.291 ± 0.01 *	79.29 ± 2.36 *
Cholestyramine (mg)	10	0.367 ± 0.02	100 ± 1.56
Cellulose (mg)	10	0.030 ± 0.01	8.17 ± 0.54

^a^, The relative bile acids binding ability of the polysaccharides was determined by the total amount of bile acids bound by it over the cholestyramine, for which its ability was defined as 100. * Statistically different from the cholestyramine (*P* < 0.05).

## Supplementary Materials

The following is available online at http://www.mdpi.com/2073-4360/11/2/263/s1, Table S1: The basic physicochemical properties of WL-CPS-1, NaCl-CPS-1 and Glu-CPS-1.
