# Supplementary material for: Comparisons of Functional Properties of Polysaccharides from Nostoc flagelliforme under Three Culture Conditions
Source: Polymers (Basel). 2019 Feb 4;11(2):263. doi: 10.3390/polym11020263 (PMC6419065; doi:10.3390/polym11020263)
Supplement: Supplementary file 1 [file polymers-11-00263-s001.pdf]

Comparisons of Functional Properties of Polysaccharides from *Nostoc flagelliforme* under Three Culture Conditions

Shi-gang Shen[1,2], Ya-hui Lin[1], Dong-xue Zhao[1], Yi-kai Wu[1], Rong-rong Yan[1], Hua-bing Zhao[1,2], Zhi-lei Tan[1, *], Shi-ru Jia[1,*], Pei-pei Han[1,*]

[1] State Key Laboratory of Food Nutrition and Safety, Key Laboratory of Industrial Fermentation Microbiology, Ministry of Education, College of Biotechnology, Tianjin University of Science and Technology, Tianjin 300457, China

[2]Tianjin Key Laboratory for Prevention and Control of Occupational and Environmental Hazards, Logistics University of Chinese People's Armed Police Forces, Tianjin 300309, China

* Corresponding author: State Key Laboratory of Food Nutrition and Safety, Key Laboratory of Industrial Fermentation Microbiology, Ministry of Education, College of Biotechnology, Tianjin University of Science and Technology, Tianjin 300457, China. Email: pphan@tust.edu.cn; jiashiru@tust.edu.cn; tanzhilei@tust.edu.cn;

Tel: +86 22 60601598; Fax: +86 22 60602298

Table S1. The basic physicochemical properties of WL-CPS-1, NaCl-CPS-1 and Glu-CPS-1.

Basic physicochemical properties		Sample name		
		WL-CPS	NaCl-CPS	Glu-CPS
Chemical composition (%)	Total sugar	80.04±2.47	83.85±3.67	85.75±3.08
	Protein	0.55±0.04	0.67±0.04	0.56±0.03
	Uronic acid	13.67±1.22	15.38±1.18	15.33±0.98
	Total polyphenols	ND	ND	ND
Average molecular weights (kDa)		1.02×10^3	1.12×10^3	1.33×10^3
Monosaccharide composition (percentage %)	Xylose	0.61±0.01	1.39±0.02	1.57±0.03
	Arabinose	0.84±0.01	1.02±0.01	1.38±0.02
	Ribose	2.39±0.04	1.90±0.01	2.95±0.03
	Rhamnose	1.47±0.03	4.97±0.02	4.69±0.02
	Fucose	0.52±0.02	1.47±0.01	1.08±0.01
	Fructose	0.09±0.01	0.36±0.03	0.09±0.01
	Mannose	19.80±0.10	20.24±0.06	20.81±0.07
	Galactose	23.71±0.05	22.62±0.21	24.34±0.05
	Glucose	46.57±0.22	41.07±0.09	39.03±0.02
	Glucuronic acid	4.00±0.08	4.97±0.01	4.04±0.02

ND, not detected
